# Clinicopathological characteristics and treatment outcome of resectable gastric cancer patients with small para-aortic lymph node

**DOI:** 10.3389/fonc.2023.1131725

**Published:** 2023-02-27

**Authors:** Zhendan Yao, Hong Yang, Ming Cui, Jiadi Xing, Chenghai Zhang, Nan Zhang, Lei Chen, Fei Tan, Kai Xu, Maoxing Liu, Xiangqian Su

**Affiliations:** Key laboratory of Carcinogenesis and Translational Research (Ministry of Education), Department IV of Gastrointestinal Cancer Center, Peking University Cancer Hospital & Institute, Beijing, China

**Keywords:** gastric cancer, para-aortic lymph node, D2 lymphadenectomy, carcinoembryonic antigen, carbohydrate antigen 19-9, prognosis

## Abstract

**Background:**

Resectable gastric cancer (GC) patients with small para-aortic lymph node (smaller than 10mm in diameter, sPAN) were seldom reported, and existing guidelines did not provide definite treatment recommendation for them.

**Methods:**

A total of 667 consecutive resectable GC patients were enrolled. 98 patients were in the sPAN group, and 569 patients without enlarged para-aortic lymph node were in the nPAN group. Standard D2 lymphadenectomy was performed. Neoadjuvant and adjuvant chemotherapy were administrated according to the cTNM and pTNM stage, respectively. Clinicopathological features and prognosis were compared between these two groups.

**Results:**

The median size of sPAN was 6 (range, 2−9) mm and the distribution was prevalent in No. 16b1. cN stage (*p*=0.001) was significantly related to the presence of sPAN. sPAN was both independent risk factor for OS (*p*=0.031) and RFS (*p*=0.046) of all patients. The prognosis of patients with sPAN was significantly worse than that of patients with nPAN (OS: *p*=0.008; RFS: *p*=0.007). Preoperative CEA and CA19-9 were independent risk factors for prognosis of patients with sPAN. Furthermore, patients in the sPAN group with normal CEA and CA19-9 exhibited acceptable prognosis (5-year OS: 67%; RFS: 64%), while those with elevated CEA or CA19-9 suffered significantly poorer prognosis (5-year OS: 17%; RFS: 17%) than patients in the nPAN group (5-year OS: 64%; RFS 62%) (both *p* < 0.05).

**Conclusions:**

Standard D2 lymphadenectomy should be considered a valid approach for GC patients with sPAN associate to normal preoperative CEA and CA19-9 levels. Patients with sPAN associated to elevated CEA or CA19-9 levels could benefit from a multimodal approach: neoadjuvant chemotherapy; radical surgery with D2 plus lymph nodal dissection extended to No. 16 station.

## Introduction

According to the revised classification of regional nodes proposed by Japanese Gastric Cancer Association, para-aortic lymph node (PAN) locates at the region from aortic hiatus to aortic bifurcation (1). Treatment strategies for GC patients with PAN metastasis have been explored for decades of years, and there are differences existed in the treatment strategies between Eastern and Western countries. In the West, PAN metastasis is identified as distant metastasis (M1), and the survival of patients with PAN metastasis is extremely poor even after extended lymph node dissection (2). In the last two decades of the 20^th^ century, oncologists from the East (especially Japan) discovered the incidence of PAN metastasis was around 20% in advanced GC patients (3, 4), and they regarded PAN as the terminal station in front of the systemic circulation, so proposed D2 lymphadenectomy plus para-aortic lymph node dissection (PAND) as the standard resection extent for advanced GC patients (5). However, the Japan Clinical Oncology Group (JCOG) 9501 trial and other Eastern countries trials demonstrated that prophylactic PAND was meaningless for locally advanced GC patients with no enlarged PAN (6, 7). Radical D2 dissection followed by adjuvant chemotherapy was recognized as the standard treatment option for locally advanced GC patients without PAN metastasis. Then, some investigators turned their attention to therapeutic PAND, and conducted a series of phase II randomized controlled trials about the effect of multidisciplinary therapy on resectable GC patients with PAN metastasis (8, 9). In these trials, they defined PAN metastasis as the node of 10mm or more in diameter around the abdominal aorta confirmed by contrast-enhanced computed tomography (CT) scan, and performed neoadjuvant chemotherapy (NAC) followed by D2 lymph node dissection with PAND. The prognosis results were encouraging (8–11). So, the Japanese gastric cancer treatment guidelines recommended that NAC combined with curative surgery as the standard treatment modality for selective GC patients with PAN metastasis (12).

So far, the treatment strategies for GC patients without PAN or with swollen PAN larger than 10mm in diameter have been basically confirmed. However, there are few reports about GC patients with PAN smaller than 10mm in diameter (sPAN). Therefore, we conducted this study to observe the clinicopathological characteristics and prognosis of resectable GC patients with sPAN.

## Materials and methods

### Patients

From April 2009 to December 2016, a total of 813 consecutive patients were diagnosed with resectable GC at the Gastrointestinal Cancer Center IV at Peking University Cancer Hospital. The inclusion criteria were as follows (1): Eastern Cooperative Oncology Group (ECOG) performance score less than 2 (2); primary gastric adenocarcinoma was confirmed by endoscopic biopsy preoperatively (3); liver, lung and Virchow lymph node metastasis were excluded by imaging examination (4); cardiopulmonary function was normal (5); preoperative liver, kidney and coagulation function were normal; and (6) no PAN larger than 10mm in diameter presented in the CT image. Exclusion criteria were as follows (1): clinical or image data incomplete (2); intraoperative laparoscopic exploration showed peritoneal implantation (3); intraoperative lavage cytology positive (4); postoperative pathological reports of neuroendocrine tumor, adenosquamous carcinoma, etc. (5); failing to perform D2 lymphadenectomy (6); history of malignant tumors. Ultimately, 667 patients in total were enrolled in this study. Written informed consent was obtained from all patients.

### Baseline assessment

**Figure 1:**
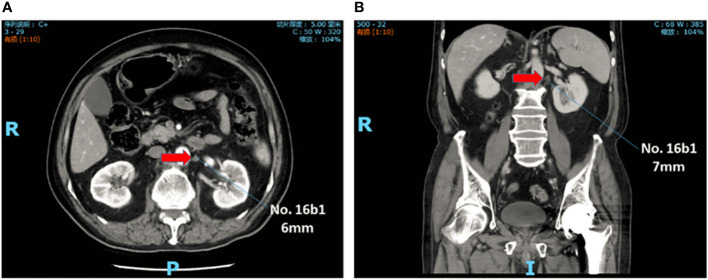
sPAN in abdominal contrast-enhanced computed tomography scan. The lymph node, with diameter less than lcm, was detected at No. 16b1. **(A)** Transverse plane. **(B)** Reconstructed image in coronal plane. sPAN, small para-aortic lymph node.

Abdominal contrast-enhanced CT scan was performed within 1 month before treatment. Skilled radiologists reviewed the images to evaluate cTNM stage. Furthermore, two experienced radiologists independently reviewed para-aortic region and reported the status and features of the PAN when it was detected. The results were presented in the form of location and size. The diameter of PAN less than 10mm was denoted as sPAN (**Figure 1**).

### Treatment strategy

Early GC patients received surgical treatment directly, and locally advanced GC patients were administrated NAC followed by instruction from multidisciplinary team (MDT) discussion firstly. The mainly NAC regimens were capecitabine plus oxaliplatin (CapeOX), S-1 plus oxaliplatin (SOX). Patients receiving NAC were reviewed every 2 cycles by contrast-enhanced CT scan to assess the response and the chance of radical surgical resection. However, it should be pointed out that, for various reasons (i.e. economic problem or mind burden), not all patients with MDT recommending NAC were treated as planned.

Once the opportunity for curative surgery was confirmed, no matter as the new-diagnosed patients or those after NAC, gastrectomy with standard D2 lymphadenectomy was performed. Ahead of gastrectomy, laparoscopic staging examination was executed to exclude patients with peritoneal plantation and/or positive results of lavage cytology. Postoperative chemotherapy started within 45 days after surgery depending on the pathological results and the recovery process. The adjuvant chemotherapy regimens included CapeOX, SOX, and S-1 monotherapy.

### Follow-up

In this study, patients were followed up every 3 months for 2 years after surgery, then every 6 months from third to fifth year. After 5 years, they were followed up once every 12 months. The median follow-up period for the cohort was 68 (range, 1-161) months, and 642 (96.3%) patients completed postoperative follow-up. The deadline of the follow-up was August 31st, 2022. Relapse-free survival (RFS) was defined as the time from the date of surgery to the first date of relapse and/or death from any cause, and overall survival (OS) was calculated from the date of surgery to death or the deadline of follow-up.

The primary end-point was 5-year OS, secondary end-points included 5-year RFS.

### Data collection

Patients’ demographic data, image examination, preoperative tumor markers [carcinoembryonic antigen (CEA), carbohydrate antigen 19-9 (CA19-9)], pathological data (tumor location, size, macroscopic type, differentiation degree, Tumor-Node-Metastasis (TNM) stage, number of lymph node harvested and number of positive), and the follow-up results were recorded in this study.

### Statistical analysis

IBM SPSS Statistics (Version 25.0; IBM Corp., New York, USA) was applied for data statistical analysis. Data are presented as the *x ± s* for continuous variables and as numbers and percentages for categorical variables. Differences between groups were calculated using the independent *t*-test, *X* ^2^ test or Fisher’s exact test, as appropriate. Cumulative RFS and OS rates were compared using the Kaplan-Meier method and Log-rank test. Cox regression model was used for multi-factor prognostic analysis. Multivariate logistic regression analysis was used for factors that were *p* < 0.1 on univariate analysis. *P* < 0.05 was considered statistically significant.

## Results

### Patients’ clinicopathological characteristics in sPAN and nPAN group

**Figure 2:**
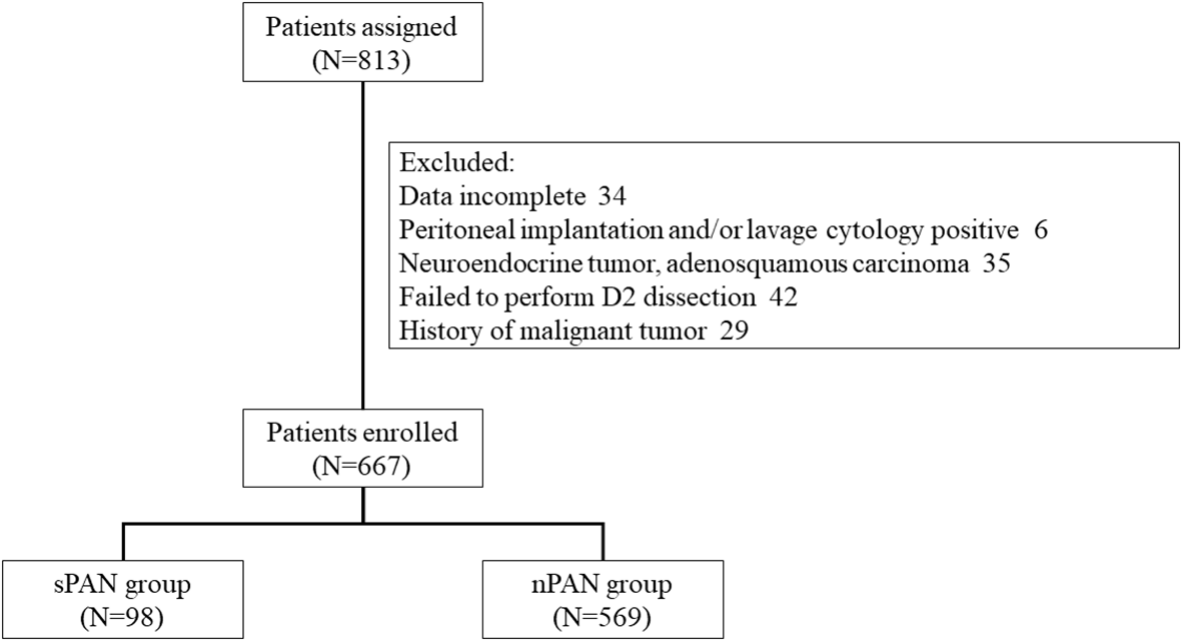
Flow diagram of treatment patterns. SPAN. small para-aortic lymph node; nPAN, non para-aortic lymph node.

From April 2009 to December 2016, 667 of 813 consecutive resectable GC patients were enrolled in this retrospective cohort study. Among them, 98 patients (14.7%) were included in the sPAN group, while the left 569 patients (85.3%), with no enlarged lymph nodes in para-aortic region, were included in the nPAN group (**Figure 2**). We performed D2 lymphadenectomy with perioperative chemotherapy (if necessary) for them.

According to the revised classification of regional nodes proposed by Japanese Gastric Cancer Association, PAN is classified into four groups, namely No. 16a1, a2, b1 and b2, from cranial to caudal. In the current study, the distribution of sPAN was 16a1 in 1 (1.0%) patients, 16a2 in 15 (15.3%), 16b1 in 65 (66.3%), 16b2 in 4 (4.1%), and cross-regional in 13 (13.3%). The median size of PAN was 6 (range, 2−9) mm.

**Table 1** shows the clinicopathological characteristics of patients in sPAN and nPAN group. No significant association was observed between PAN status and age, pathological T (pT) stage, pathological TNM (pTNM) stages, preoperative CEA and CA19-9 level, tumor location, tumor size, differential grade (*p*>0.05). The proportion of male (80.6% vs 69.9%, *p*=0.031), macroscopic type 3/4 (74.5% vs 60.5%, *p*=0.008) and number of metastatic lymph nodes (6.0 ± 8.3 vs 3.9 ± 5.9, *p*=0.017) were significantly higher in sPAN group than in nPAN group (*p*<0.05). Besides, there were more patients with advanced clinical T (cT) stage and clinical N (cN) stage in the sPAN group than that in the nPAN group (*p*<0.05). Moreover, 15 of 98 (15.3%) patients received NAC in the sPAN group compared with 35 of 569 (6.2%) patients in the nPAN group (*p*=0.001).

**Table 1:** Clinicopathological characteristics of patients in sPAN and nPAN group.

Clinicopathological characteristics	sPAN (N=98) (%)	nPAN (N=596) (%)	Statistics value	*p*
Male, n (%)	79 (80.6)	398 (69.9)	*X* ^2^=4.668	0.031
Age (year, mean ± SD)	57.9 ± 12.3	59.0 ± 10.8	*t*=0.821	0.413
NAC, n (%)	15 (15.3)	35 (6.2)	*X* ^2^=10.105	0.001
CEA elevated, n (%)	19 (19.4)	98 (17.2)	*X* ^2^=0.271	0.603
CA19-9 elevated, n (%)	15 (15.3)	68 (12.0)	*X* ^2^=0.864	0.353
Tumor location, n (%)			*X* ^2^=0.580	0.446
Upper	26 (26.5)	154 (27.1)		
Middle	30 (30.6)	176 (30.9)		
Low	27 (27.6)	178 (31.3)		
Total	15 (15.3)	61 (10.7)		
Macroscopic type, n (%)			*X* ^2^=7.026	0.008
1+2	25 (25.5)	225 (39.5)		
3+4	73 (74.5)	344 (60.5)		
Tumor size (cm, mean ± SD)	4.47 ± 2.59	4.24 ± 2.45	*t*=0.847	0.397
Differential grade, n (%)			*X* ^2^=0.799	0.371
Well	29 (29.6)	144 (25.3)		
Poorly	69 (70.4)	425 (74.7)		
cT stage, n (%)			*X* ^2^=6.081	0.014
cT1+2	13 (13.3)	140 (24.6)		
cT3+4	85 (86.7)	429 (75.4)		
cN stage, n (%)			*X* ^2^=18.257	<0.001
cN0	49 (50)	408 (71.7)		
cN+	49 (50)	161 (28.3)		
pT stage, n (%)			*X* ^2^=2.918	0.404
T1	10 (10.2)	93 (16.3)		
T2	18 (18.4)	92 (16.2)		
T3	45 (45.9)	231 (40.6)		
T4	25 (25.5)	153 (26.9)		
pN stage, n (%)			*X* ^2^=16.447	0.001
N0	39 (39.8)	309 (54.3)		
N1	26 (26.5)	115 (20.2)		
N2	17 (17.3)	110 (19.3)		
N3	16 (16.3)	35 (6.2)		
pTNM stage, n (%)			*X* ^2^=2.306	0.316
I	19 (19.4)	141 (24.8)		
II	31 (31.6)	191 (33.6)		
III	48 (49.0)	237 (41.7)		
Harvested LNs (mean ± SD)	32.3 ± 13.2	30.5 ± 16.2	*t*=1.203	0.231
Metastatic LNs (mean ± SD)	6.0 ± 8.3	3.9 ± 5.9	*t*=2.415	0.017

sPAN, small para-aortic lymph node; nPAN, non para-aortic lymph node; NAC, neoadjuvant chemotherapy; CEA, carcinoembryonic antigen; CA19-9, carbohydrate antigen 19-9; LN, lymph node.

### Risk factors for sPAN

In the univariable analysis of risk factors for sPAN, gender (*p*=0.031), NAC (*p*=0.001), macroscopic type (*p*=0.044), cT stage (*p*=0.014), cN stage (*p*<0.001), and pathological N (pN) stage (*p*=0.001) were significantly associated with the presence of sPAN.

In the multivariable analysis of the risk factors for sPAN, cN stage (*p*=0.001) was significantly related to the presence of sPAN (**Table 2**).

**Table 2:** Preoperative risk factors of sPAN by logistic regression analysis.

Factors	B	SE	Wald	df	Exp(B)	95% CI	*p*
Gender	0.426	0.278	2.352	1	1.532	0.888-2.641	0.125
Macroscopic type	-0.364	0.267	1.863	1	0.695	0.412-1.172	0.172
cT stage	-0.275	0.343	0.643	1	0.760	0.388-1.487	0.423
cN stage	-0.747	0.230	10.543	1	0.474	0.302-0.744	0.001
pN stage	-0.194	0.109	3.160	1	0.824	0.665-1.020	0.075

sPAN, small para-aortic lymph node; SE, standard error; CI, confidence interval.

### Survival analysis of all GC patients

The median OS of all GC patients was 68 (IQR 37~91) months, and the 1-, 3- and 5-year OS rates were 84%, 71% and 62%, respectively. Univariate Cox regression analysis showed that age, CEA, CA19-9, macroscopic type, tumor size, cT stage, cN stage, PAN status, differential grade, pT stage, pN stage, pTNM stage were correlate with OS of all patients (*p*<0.05). The above factors, plus other factors with *p*<0.1, were selected as covariables in the multivariate Cox regression model. The results revealed that age (*p*<0.001), CA19-9 (*p*=0.014), PAN status (*p*=0.031), tumor size (*p*=0.031), pT stage (*p*<0.001), pTNM stage (*p*=0.013) were independent risk factors for OS of all patients (**Table 3**).

**Table 3:** Univariate and multivariate analysis of risk factors for OS and RFS of overall gastric cancer patients.

Clinicopathological characteristics	OS				RFS			
	Univariate analysis		Multivariate analysis		Univariate analysis		Multivariate analysis	
	HR (95% CI)	*P*	HR (95% CI)	*P*	HR (95% CI)	*P*	HR (95% CI)	*P*
Gender								
Male	1				1			
Female	0.780 (0.592-1.029)	0.078	–		0.779 (0.595-1.021)	0.071	–	
Age (years)								
<=65	1		1		1		1	
>65	1.637 (1.273-2.106)	<0.001	1.621 (1.250-2.103)	<0.001	1.518 (1.185-1.944)	0.001	1.489 (1.154-1.922)	0.002
CEA (ng/ml)								
<5	1		1		1			
>= 5	1.384 (1.027-1.866)	0.033	0.951 (0.694-1.303)	0.755	1.300 (0.966-1.750)	0.083	–	
CA19-9 (u/ml)								
<37	1		1		1		1	
>= 37	2.270 (1.672-3.082)	<0.001	1.484 (1.082-2.035)	0.014	2.037 (1.499-2.767)	<0.001	1.319 (0.961-1.811)	0.087
Macroscopic type								
1-2	1		1		1		1	
3-4	2.077 (1.579-2.733)	<0.001	1.031 (0.742-1.433)	0.857	1.977 (1.517-2.577)	<0.001	1.027 (0.746-1.413)	0.872
Tumor size (cm)								
<5	1		1		1		1	
>= 5	2.201 (1.731-2.799)	<0.001	1.365 (1.027-1.815)	0.032	2.121 (1.676-2.684)	<0.001	1.349 (1.021-1.783)	0.035
Differential grade								
well	1		1		1		1	
Poorly	1.555 (1.153-2.096)	0.004	1.150 (0.830-1.593)	0.401	1.490 (1.116-1.990)	0.007	1.098 (0.800-1.507)	0.564
PAN status								
sPAN	1		1		1		1	
nPAN	0.662 (0.488-0.899)	0.008	0.706 (0.514-0.970)	0.032	0.667 (0.495-0.900)	0.008	0.728 (0.533-0.994)	0.046
cT stage								
cT1-2	1		1		1		1	
cT3-4	3.313 (2.225-4.933)	<0.001	0.943 (0.599-1.483)	0.799	2.891 (2.000-4.179)	<0.001	0.897 (0.588-1.368)	0.613
cN stage								
cN0	1		1		1		1	
cN+	1.616 (1.263-2.066)	<0.001	1.184 (0.916-1.531)	0.197	1.546 (1.214-1.969)	<0.001	1.149 (0.893-1.478)	0.280
pT stage								
T1	1		1		1		1	
T2-4	2.198 (1.888-2.557)	<0.001	1.658 (1.313-2.094)	<0.001	2.109 (1.823-2.441)	<0.001	1.593 (1.272-1.996)	<0.001
pN stage								
N0	1		1		1		1	
N1-3	1.489 (1.328-1.670)	<0.001	1.110 (0.961-1.282)	0.155	1.475 (1.318-1.651)	<0.001	1.107 (0.961-1.275)	0.158
pTNM stage								
I	1		1		1		1	
II-III	2.746 (2.269-3.323)	<0.001	1.499 (1.089-2.062)	0.013	2.621 (2.184-3.146)	<0.001	1.519 (1.115-2.070)	0.008

OS, overall survival; RFS, relapse free survival; HR, hazard ratio; CI, confidence interval; CEA, carcinoembryonic antigen; CA19-9, carbohydrate antigen 19-9; PAN, para-aortic lymph node; nPAN, non para-aortic lymph node; cT, clinical T; cN, clinical N; pT, pathological T; pN, pathological N; pTNM, pathological tumor-node-metastasis.

The median RFS of all patients was 65 (IQR 27~89) months, and the 1-, 3-, 5-year RFS rates were 78%, 66%, and 60%, respectively. Univariate analysis revealed that age, CA19-9, macroscopic type, cT stage, cN stage, PAN status, differential grade, pT stage, pN stage, pTNM stage were correlate with RFS of all patients (*p*<0.05). The above variables, together with other variables with *p*<0.1, were enrolled in the multivariate analysis. And the results indicated that age (*p*=0.002), PAN status (*p*=0.046), pT stage (*p*<0.001), pTNM stage (*p*=0.008) were independent risk factors for RFS (**Table 3**).

### Comparison of prognosis between sPAN and nPAN group

**Figure 3:**
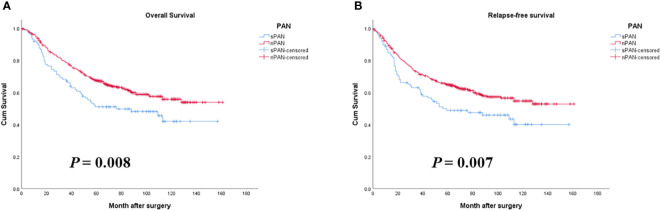
Survival curves for GC patients with SPAN and nPAN. **(A)** Patients with SPAN had worse OS than patients with nPAN (*p* = 0.008); **(B)** Patients with SPAN had worse RFS than those with nPAN (*p* = 0.007). PAN, para-aortic lymph node; sPAN, small para-aortic lymph node; nPAN, non para-aortic lymph node; GC, gastric cancer; OS, overall survival; RFS, relapse-free survival.

The 1-, 3- and 5-year OS rates in the sPAN group and the nPAN group were 76% *vs*. 85%, 60% *vs*. 73% and 51% *vs*. 64%, respectively (*p*=0.008) (**Figure 3A**). Meanwhile, the 1-, 3- and 5-year RFS rates in the sPAN group and the nPAN group were 65% *vs*. 80%, 54% *vs.* 68% and 49% *vs*. 63%, respectively (*p*=0.007) (**Figure 3B**). Kaplan-Meier analysis revealed that both the OS and RFS of patients in the sPAN group were significantly worse than that of patients in the nPAN group.

### Survival analysis of patients in the sPAN group

The univariate and multivariate COX regression analyses for patients in the sPAN group were summarized in **Table 4**.

**Table 4:** Univariate and multivariate analysis of risk factors for OS and RFS of patients in the sPAN group.

Clinicopathological characteristics	OS				RFS			
	Univariate analysis		Multivariate analysis		Univariate analysis		Multivariate analysis	
	HR (95% CI)	*P*	HR (95% CI)	*P*	HR (95% CI)	*P*	HR (95% CI)	*P*
Gender								
Male	1		–		1		–	
Female	0.661 (0.310-1.407)	0.283			0.745 (0.364-1.528)	0.422		
Age (years)								
<=65	1		–		1		–	
>65	1.464 (0.817-2.621)	0.200			1.284 (0.721-2.286)	0.396		
CEA (ng/ml)								
<5	1		1		1		1	
>= 5	3.162 (1.728-5.788)	<0.001	3.198 (1.686-6.066)	<0.001	2.794 (1.538-5.077)	0.001	2.646 (1.420-4.933)	0.002
CA19-9 (u/ml)								
<37	1		1		1		1	
>= 37	4.025 (2.146-7.550)	<0.001	3.051 (1.544-6.029)	0.001	3.561 (1.908-6.644)	<0.001	2.703 (1.371-5.331)	0.004
Macroscopic type								
1-2	1		1		1		1	
3-4	2.298 (1.078-4.900)	0.031	1.168 (0.471-2.894)	0.738	2.143 (1.044-4.400)	0.038	1.137 (0.473-2.731)	0.774
Tumor size (cm)								
<5	1		1		1		1	
>= 5	1.875 (1.078-3.260)	0.026	1.125 (0.606-2.087)	0.710	1.929 (1.121-3.320)	0.018	1.249 (0.678-2.300)	0.475
Differential grade								
well	1		–		1		–	
Poorly	1.111 (0.608-2.032)	0.731			1.256 (0.690-2.287)	0.455		
cT stage								
cT1-2	1		1		1		1	
cT3-4	5.217 (1.267-21.478)	0.022	1.086 (0.211-5.582)	0.921	3.519 (1.096-11.292)	0.034	0.894 (0.219-3.643)	0.876
cN stage								
cN0	1		–		1		–	
cN+	1.452 (0.837-2.520)	0.185			1.423 (0.829-2.445)	0.201		
pT stage								
T1	1		1		1		1	
T2-4	2.352 (1.604-3.448)	<0.001	1.826 (1.130-2.950)	0.014	2.056 (1.432-2.952)	<0.001	1.598 (1.006-2.538)	0.047
pN stage								
N0	1		1		1		1	
N1-3	1.339 (1.051-1.705)	0.018	1.186 (0.881-1.596)	0.260	1.334 (1.052-1.690)	0.017	1.196 (0.893-1.601)	0.230
pTNM stage								
I	1		1		1		1	
II-III	2.587 (1.651-4.053)	<0.001	1.261 (0.643-2.476)	0.500	2.314 (1.519-3.524)	<0.001	1.205 (0.627-2.313)	0.576

OS, overall survival; RFS, relapse free survival; sPAN, small para-aortic lymph node; HR, hazard ratio; CI, confidence interval; CEA, carcinoembryonic antigen; CA19-9, carbohydrate antigen 19-9; cT, clinical T; cN, clinical N; pT, pathological T; pN, pathological N; pTNM, pathological tumor-node-metastasis.

Based on univariate analysis, the risk factors that might affect OS of patients in the sPAN group with *p* < 0.05 (CEA, CA19-9, macroscopic type, tumor size, cT stage, pT stage, pN stage, pTNM stage) were chosen to performed multivariate analysis. Subsequently, the results of multivariate analysis revealed that elevated CEA (*p*<0.001), elevated CA19-9 (*p*=0.001), advanced pT stage (*p*=0.014) were independent poor survival factors for OS of patients in the sPAN group.

Univariate analysis indicated that CEA, CA19-9, macroscopic type, cT stage, pT stage, pN stage, and pTNM stage were associated with RFS of patients in the sPAN group (*p*<0.05). All factors above were included in the multivariate Cox regression analysis. The results showed that elevated CEA (*p*=0.002), elevated CA19-9 (*p*=0.005) were independent poor survival factors for RFS of patients in the sPAN group (**Table 4**).

Particularly, univariate analysis indicated that the size and location of sPAN were not correlated with OS or RFS of patients in the sPAN group (*p* > 0.05).

### Risk factors of prognosis in sPAN group

**Figure 4:**
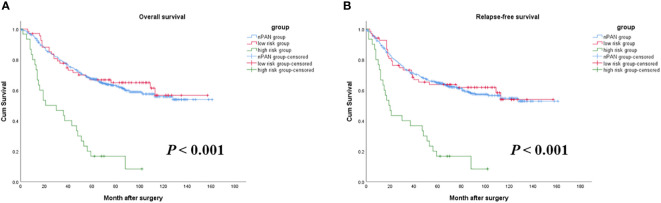
Survival curves for SPAN patients with low risk, high risk and nPAN. **(A)** sPAN patients with high risk had much worse OS than those with low risk and nPAN (*p* < 0.001); **(B)** sPAN patients with high risk had much worse RFS than those with low risk and nPAN (*p* < 0.001). sPAN, small para-aortic lymph node; nPAN, non para-aortic lymph node; OS, overall survival; RFS, relapse-free survival; GC, gastric cancer.

As mentioned above, preoperative elevated CEA and CA19-9 level were both relevant to worse OS and RFS in the sPAN group. Then, patients in the sPAN group were divided into two groups, namely low-risk group (both CEA and CA19-9 were normal) and high-risk group (at least one of the tumor markers was abnormal). There were 68 cases in low-risk group, and 30 cases in high-risk group. The differences in postoperative survival among the above two groups, as well as the nPAN group were compared together. The results revealed no significant differences existed in OS or RFS between low-risk group (5-year OS: 67%; RFS: 64%) and nPAN group (5-year OS: 64%; RFS 62%), while the prognosis in high-risk group (5-year OS: 17%; RFS: 17%) was significantly inferior to the former two groups (*p* < 0.05) (**Figure 4**).

### Postoperative recurrence of PAN

During follow-up, para-aortic region recurrences were found in 17 (2.5%) patients. There were 3 cases (3.1%) in the sPAN group, and 14 cases (2.5%) in the nPAN group. The median time interval for PAN recurrence was 12 (range, 2−19) months and 22 (range, 8−50) months in the sPAN and nPAN group, respectively. However, the differences were not statistically significant (*p >* 0.05).

## Discussion

GC patients with sPAN are a special entity. Due to the deep location and continuous beating of the aorta nearby, it is difficult and risky to biopsy, so obtaining pathological result of sPAN before treatment is of great challenge. Some other alternative diagnosis tools, such as CT, endoscopic ultrasound (EUS), and positron emission tomography (PET), have been applied for detecting the status of lymph nodes in gastric cancer (13). However, the small size of sPAN makes false positive results inevitably. It is unreasonable to simply classify the sPAN in to inflammatory or metastatic nodes. So far, the clinical features and prognosis of GC patients with sPAN are rarely discussed, and existing guidelines do not provide definite treatment recommendation for such patients.

Our study discovered the proportion of male, macroscopic type 3/4, clinical T3/4, clinical N+ and number of metastatic lymph nodes were obviously high in the sPAN group, and multivariate analysis demonstrated that clinical N stage was significantly associated with the presence of sPAN. Therefore, for GC patients with clinical N+, special attention should be paid to the evaluation of sPAN.

In the present study, PAN status was independent risk factors for OS and RFS of overall patients (*p*<0.05). Moreover, the rate of 5-year OS and RFS of GC patients in the sPAN group was both significantly worse than those of patients in the nPAN group (51% vs 64%, *p*=0.008; 49% vs 63%, *p*=0.007, respectively). We suspected this may be due to two reasons. Firstly, we enrolled patients with PAN smaller than 10mm in diameter in this study. In fact, this criterion would not exclude patients with PAN metastasis effectively. In the study of Lee JH, et al. (14), the authors focused on the advanced GC patients with equivocal findings of PAN metastasis (nodes size from 7~10mm or larger than 10mm with a fatty marrow) on CT image. All patients were performed D2 gastrectomy plus PAND, and histopathological results showed that 10/23 (43.5%) patients had PAN metastasis. In our study, the median size of PAN was 6 (range, 2-9) mm, which meant that our patients should share several similar clinicopathological characteristics to those in Lee’s study. And it was reasonable that some patients with PAN metastasis, mixed into the sPAN group. This subset of patients might be detected early before their PAN had grown large enough. Although the PAN was smaller than 10mm in size, the node was metastatic in nature, and therefore the patient had a poor prognosis. The incorporation of these patients finally worsened survival rate in the sPAN group.

Secondly, it was not until 2018 that NAC was formally recommended in the Japanese gastric cancer treatment guidelines (5th edition) (15). So, when our study was conducted in 2009, NAC for GC was not widely recognized in the East. Whether to receive NAC depended on the clinical TNM stage and the treatment intent of patient. Although the incidence of NAC in the sPAN group was significantly higher than that in the nPAN group (15.3% vs 6.2%, *p*=0.001), the actual rate of NAC in our study was still low. Recently, oncologists around the world generally recognized that NAC played an increasingly important role in the treatment of advanced GC (16–19). Studies from China revealed that NAC could significantly reduce the size of the PAN in some patients with PAN metastasis (20). They performed D2 dissection after the size of PAN shank to < 10mm, and reported the survival of patients received surgery was significantly improved, even comparable to that of the JCOG trials. The authors concluded that NAC could cleared the occult metastasis in the PAN, and then patients achieved long-term survival without PAN dissection. Therefore, it could be cautiously inferred that the low incidence of NAC in our study might be associated with the poor prognosis of patients in the sPAN group.

One of the unsolved questions is whether the survival results of patients in the sPAN group could be improved by expanding the extent of lymph node dissection. Although the present study could not provide definitive answers, it still offered some meaningful suggestions. We found both patients in the sPAN and nPAN group had a low rate of recurrence at the PAN region after radical D2 dissection, and the difference was not statistically significant. So PAND itself could not reduce the recurrent rate for patients with sPAN efficiently. At the same time, even for skilled operators, the peri- and post-operative complication rates of PAND were still high (21–23). So, it is inferred that PAND may be a high-risk treatment option with low benefit for GC patients with sPAN.

Historically, in some studies, a diameter of 8mm or 6mm was used as the criteria for positive lymph node in GC patients (24–26). Unfortunately, most of the studies reported high false-positive rates (27). A higher false-positive rate meant more patients with enlarged PAN were regarded as PAN metastases. Actually, GC patients with PAN metastasis in our center were referred to the Digestive Oncology Department for systemic chemotherapy. Thus, they might miss the opportunity for surgery. Therefore, at the beginning of the study design, the diameter of 10mm was selected as the criterion for evaluate PAN status. In our view, this had two major advantages: firstly, the inclusion criteria of this study were consistent with those of previous JOCG studies, which made it easier to compare the prognosis of patients; secondly, reducing the false-positive rate made more patients had the opportunity to obtain radical surgery.

However, everything had its own two sides. Loosening the inclusion criteria inevitably enrolled patients with poor prognosis. In fact, sPAN could be caused by benign lymph node hyperplasia or malignant metastasis, but there was no absolute criterion that completely distinguished the metastatic from non-metastatic PAN. One effective way was to establish a relatively simple and feasible method to predict patients with poor prognosis in the sPAN group. We noticed that preoperative CEA and CA19-9 levels were independent risk factors for the prognosis of patients with sPAN, and these two items were routinely performed preoperatively recommended by the National Comprehensive Cancer Network (NCCN) guidelines. Therefore, we used them as a screening protocol and classified sPAN patients into the two subgroups, that was low-risk sPAN group (normal preoperative CEA and CA19-9 levels) and high-risk sPAN group (elevated levels of either or both of CEA and CA19-9). We compared prognosis of patients in low-risk sPAN group, high-risk sPAN group and nPAN group together, and found this screening protocol was efficient because it could effectively distinguish two categories of patients with totally different prognosis. The survival difference was not significant between patients with low-risk sPAN and nPAN, while the survival of patients in high-risk sPAN group was significantly worse than the other two groups.

Several studies revealed that preoperative CEA and CA19-9 levels could predict lymph node metastasis of GC patients. Ding BC, et al. discovered that higher level of CEA (OR 1.447, 95%CI 1.046-2.002) and CA19-9 (OR 1.529, 95%CI 1.151-2.029) were associated with a higher risk of lymph node metastasis (28). Wang K, et al. reported that preoperative serum CEA (OR 4.86, 95%CI 2.33-10.139) was significantly associated with positive lymph node metastasis (29). Feng F, et al. also found that the level of CA19-9 was correlated with the presence of lymph node metastasis for early GC patients (30). Moreover, many studies have indicated that preoperative serum CEA and CA19-9 levels are independent risk factors for prognosis and recurrence in patients with gastric cancer. Abdullah Sisik, et al. showed that positive levels of both CEA and CA19-9 could indicate GC patients in advanced stage (31). Zhou YC, et al. observed 1075 consecutive GC patients in a single tertiary hospital, and discovered both CEA and CA19-9 positively correlated with several clinicopathologic features including pTNM stage (32). Kambara Y, et al. reported that the prognosis of patients with CA19-9 > 46.3 U/ml were significantly poorer than those CA19-9 < 46.3 U/ml in stage III GC (33). Currently, it is generally believed that CEA and CA19-9 play an important role in the screening, diagnosis, prognosis assessment and recurrence prediction of GC patients. Besides, CEA and CA19-9 correlated significantly with serum levels of intercellular adhesion molecules, and the high level reflected aggressive invasive potential (34). So, it was reasonable that patients in the sPAN group with high level of CEA and/or CA19-9 exhibited extremely poor prognosis results.

In the JCOG 9501 trial (6), after excluding GC patients with enlarged and/or hard PAN, the 5-year OS and RFS rate was 69.2% and 62.6% for the patients assigned to D2 gastrectomy alone, and 70.3% and 61.7% for the patients assigned to D2 gastrectomy plus PAND, respectively. The differences were not statistically significant (*p*=0.57 for OS, and *p*=0.72 for RFS, respectively). The authors concluded that D2 gastrectomy was adequate for curable GC patients without PANM. In our study, the 5-year OS and RFS rate was 67% and 64% of the patients in the low-risk sPAN group. The result was both comparable to the prognosis of patients in the nPAN group (64% for 5-year OS rate, and 63% for 5-year RFS rate, respectively), and to that in the JCOG 9501 trial. Then, we confirmed that D2 gastrectomy should be suitable for GC patients in the sPAN group with normal preoperative CEA and CA19-9 levels.

Survival analysis showed that median survival time and median relapse-free time were 25.5 months and 14.5 months for patients in the high-risk sPAN group, respectively. The 5-year OS rate and RFS rate was both 17%. This prognosis was similar to that of patients with PAN metastasis treated by D2 lymphadenectomy plus PAND (6, 35, 36). Although we had no pathological evidence to prove that high-risk sPAN was equivalent to PAN metastasis, the fact that GC patients in the high-risk sPAN group suffered extremely poor prognosis suggested that the treatment strategy of D2 lymphadenectomy plus perioperative chemotherapy (if necessary) should not be indicated for them.

There were several limitations of this study. Firstly, it was a single-arm, retrospective design. Secondly, the patients enrolled in this study did not represent all GC patients with sPAN, but only those who underwent radical D2 lymph node dissection. Thirdly, the population of patients with sPAN was relatively small, resulting in only 2 cases with high risk in the sPAN group recurrent to PAN after surgery, so it was impossible to further analyze their clinicopathological features and survival. A prospective trial is needed to make a definitive conclusion about the optimal therapeutic strategy for GC patients in the sPAN group with elevated preoperative CEA and CA19-9 levels.

## Conclusion

Standard D2 lymphadenectomy should be considered a valid approach for GC patients with sPAN associate to normal preoperative CEA and CA19-9 levels. Patients with sPAN associated to elevated CEA or CA19-9 levels could benefit from a multimodal approach: neoadjuvant chemotherapy; radical surgery with D2 plus lymph nodal dissection extended to No. 16 station.

## Data availability statement

The raw data supporting the conclusions of this article will be made available by the authors, without undue reservation.

## Ethics statement

The studies involving human participants were reviewed and approved by Peking University Cancer Hospital Ethics Committee. The patients/participants provided their written informed consent to participate in this study.

## Author contributions

ZY, HY, XS designed the study. LC, FT collected the data. ZY, HY, KX, ML performed the statistical analysis. ZY drafted the manuscript. MC, JX, CZ, NZ, XS critically revised the manuscript for important content. All authors contributed to the article and approved the submitted version.
